# EAF2: a tumor suppressor gene with multi-aspect functions

**DOI:** 10.3389/fphar.2024.1440511

**Published:** 2024-11-11

**Authors:** Wen-Tong Ji, Chun-Guo Cui, Yao Wang

**Affiliations:** ^1^ Urology 2nd Department, China-Japan Union Hospital of Jilin University, Changchun, Jilin, China; ^2^ Galactophore Department, China-Japan Union Hospital of Jilin University, Changchun, Jilin, China; ^3^ Jilin Key Laboratory of Molecular Diagnosis of Urologic Neoplasms, Urology 2nd Department, China-Japan Union Hospital of Jilin University, Changchun, Jilin, China

**Keywords:** EAF2, tumor suppressor, transcription factor, prostate cancer, tumorigenesis

## Abstract

Since ELL-associated factor 2 (EAF2) was identified in 1997 as an androgen response gene, it has been of medical and scientific interest. Early studies demonstrated the tumor-suppressing function of EAF2 in the prostate. Sequencing studies indicated an association between EAF2 and several other malignant diseases and multiple physiological processes, such as transcription, apoptosis, embryogenesis, and DNA repair. Further understanding of EAF2 will provide new opportunities and therapeutic approaches for cancers, especially prostate cancer. This narrative review summarizes the existing knowledge of EAF2 and outlines its potential significance. To our knowledge, this is the first review of the role of this novel tumor suppressor gene and its possible functions.

## 1 Introduction

ELL-associated factor 2 (EAF2) is a binding partner of ELL family proteins (Simone et al., 2003) and an androgen-response tumor suppressor (Xiao et al., 2003; Pascal et al., 2018; Wang et al., 2017). Studies on *EAF2* have been ongoing for over 26 years, gaining significant findings regarding the functions of this gene. We found that this gene is regulated by androgens (Xiao et al., 2003) and can interact with multiple genes by binding to protein products (Pascal et al., 2018). Besides, EAF2 is possibly associated with apoptosis, embryogenesis, and DNA repair through a network of pathways (Wang et al., 2017; Zhuang et al., 2008; Hahn et al., 2007). Most importantly, EAF2 is a tumor suppressor gene, and its downregulation and deficiency correlate with the occurrence and progression of multiple tumors. The role of EAF2 in leukemia, prostate cancer, colorectal cancer, glioblastoma, and gastric cancer has been identified (Zhuang et al., 2008; Xiao et al., 2008; Sun et al., 2020).

Several EAF2 studies have focused on prostate cancer. The androgen receptor (AR) and its signaling pathways play central roles in the proliferation, migration, invasion, and differentiation of prostate cancer cells (Bruchovsky et al., 1975; Aurilio et al., 2020). Therefore, understanding the close relationship between EAF2 and androgens would result in the exploration of the pathophysiology of prostate cancer and possible treatments. As the associated factors of EAF2, such as hypoxia-induced factor 1α (HIF-1α), PTEN, von Hippel–Lindau protein (pVHL), and p53, were identified in sequence, the network of the downstream pathways of EAF2 in prostate cancer would be gradually completed.

The function of EAF2 was also reported in other physiological processes and organs. For instance, amino acids 68–113 of EAF2 were capable of inducing apoptosis in human LNCaP cells and EAF2 could cooperate with ELL in the induction of apoptosis (Hahn et al., 2007). EAF2 with intact amino acids 163–262 could work with Ku70/Ku80 in repairing DNA damage (Ai et al., 2017). EAF2 was also observed to affect convergence and extension movements (Lumetti et al., 2014; Liu et al., 2009) and eye development in embryogenesis (Wan et al., 2010) through the non-canonical Wnt/β-catenin signaling pathway. The extension of the function of EAF2 not only leads to a better understanding of development but may also shed light on how the tumor suppressor contributes to tumor initiation and progression. In the present review, we detail, for the first time, what is known about the tumor suppressor gene, its relationships with different diseases, and possible functions in multiple physiological processes.

## 2 Identification of EAF2

EAF2, previously called U19, was first identified as an androgen-responsive gene in the rat prostate in 1997 by Wang et al. (1997). In a study using castrated rats to explore androgen response gene expression, 26 genes were upregulated by androgens, and four were downregulated. U19 was the 19th gene isolated from the list of upregulated genes by Wang et al.; however, U19 was not specifically studied at that time.

In 2003, U19 was reported independently and simultaneously by two researchers from two perspectives. Xiao et al. extended the research by Wang et al. and explored the relationship between androgens and U19. The results of their experiments about U19 also suggested the possible function of tumor suppression and growth inhibition for the first time (Xiao et al., 2003). Simone et al. reported EAF2 after its homologue EAF1. Originally, Simone was trying to delve into novel proteins associated with the eleven-nineteen lysine-rich leukemia gene (ELL), an RNA polymerase II elongation factor. After EAF1 was first identified by his team, they soon isolated the second one, which was highly homologous to EAF1, and named it EAF2 (Simone et al., 2003). Xiao and Simone quickly realized that U19 and EAF2 were identical. Their subsequent cooperative study reported the important role of ELL in EAF2-induced growth suppression and apoptosis, highlighted the potential effect of transcription activating, and revealed an interesting interacting transactivation domain in the C-terminus of EAF2, which made EAF2 gradually become a research focus and vigorously promoted following researches (Hahn et al., 2007).

## 3 EAF2 protein

**FIGURE 1:**
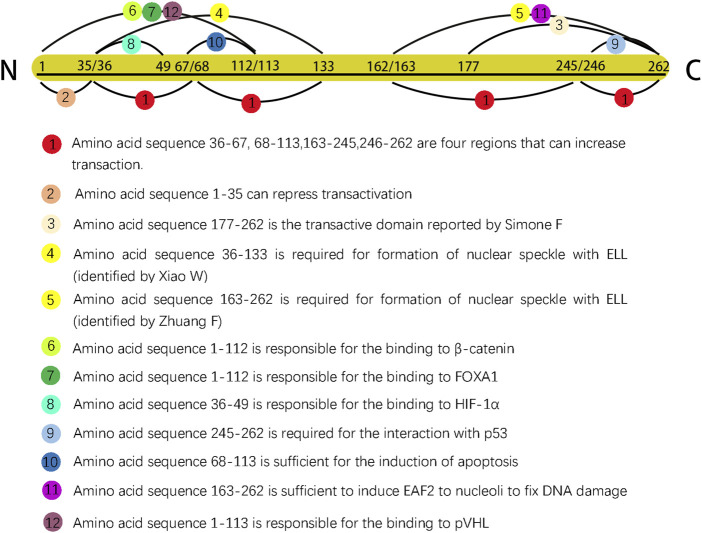
Different sequences in EAF2 are represented by dots of various colors. Dots 4 and 5 are shown in yellow color to indicate that the two sequences were identified by two separate groups as having the same function. The length ratio of segments did not represent actual ratio.

EAF2 was initially reported to contain 260 amino acids and an approximate isoelectric point of 4.84; similar to its homologue EAF1, EAF2 is rich in serine, aspartic, and glutamic acid residues (Simone et al., 2003). In 2007, after conducting serial truncations of EAF2, Zhuang et al. identified amino acid 262 as the last residue in the C-terminal domain (Zhuang et al., 2008). EAF2 with 260 and 262 amino acids could both exert biological functions fully and completely. The discrepancy of the last amino acid of C-terminal was due to the experimental design of various research groups. In order to give a more complete picture of EAF2 functionally and structurally, we summarized research findings in Figure 1 (no.262 as the last animo acid).

### 3.1 EAF2 is an unstable nuclear protein

Previous studies have observed that ELL co-localizes and interacts with EAF2 in nuclear speckles to increase RNA polymerase II elongation activity and serve as a transcription regulator (Xiao et al., 2006; Kong et al., 2005). In order to confirm the previous findings and clarify the intracellular trafficking of EAF2, Zhuang et al. transfected the GFP-EAF2 fusion protein into C2C12 myoblast cells and continuously recorded GFP fluorescence (Zhuang et al., 2008). Compared to the fluorescence signal of GFP in the control experiment, the signal of the GFP-EAF2 fusion protein became dimmer more quickly and finally disappeared after 48 h, suggesting that EAF2 is an unstable nuclear protein; however, a ring of GFP-EAF2 fluorescence was detected in the cytoplasm surrounding the nucleus when the signal was barely detectable, suggesting that a potential negative feedback mechanism may be activated after the nuclear accumulation of EAF2. Next, several truncations of EAF2 were transfected into C2C12 myoblast cells to determine the domain responsible for the nuclear import of EAF2. They found that amino acids 1–163 at the N-terminus of EAF2 showed clear nuclear localization, suggesting that this protein domain is required for nuclear import.

### 3.2 EAF2 protein undergoes polyubiquitination

The ubiquitin-proteasome system regulates ubiquitination, which involves the attachment of ubiquitin to substrate proteins for degradation. EAF2 undergoes ubiquitination, with lysine residue K81 identified as the most critical site for polyubiquitination (Yu et al., 2016). This polyubiquitination is catalyzed by SIAH2, an E3 ligase, and inhibited by ELL1. SIAH2 has been reported to be upregulated in castration-resistant prostate cancer (Hahn et al., 2007), suggesting that its role in facilitating EAF2 degradation may be one of the underlying mechanisms. In contrast, ELL1 can bind to EAF2 and block EAF2 polyubiquitination, indicating that ELL significantly enhances EAF2 stability. This is consistent with the interaction between ELL and EAF2.

### 3.3 The distribution of EAF2 protein is spatially regulated

Murine EAF2 contains remarkable amino acid conservation with human EAF2; therefore, Li et al. isolated EAF2 from adult mice and found that EAF2 expression is higher in organs that possess reproductive functions, such as the testis, ovary, uterus, mammary glands, and organs whose development requires reciprocal induction between the epithelium and mesenchyme, such as the teeth, vibrissae follicles, mammary placodes, kidneys, lungs, and pancreas (Li et al., 2003). In humans, the role of EAF2 in most of the abovementioned organs has not yet been explored; EAF2 in the prostate has been the main focus.

## 4 EAF2 functions as a transcription factor and mediates transcriptional activity

According to the definition, transcription factor (TF) has been used to describe any protein involved in transcription and/or capable of altering gene expression by (1) binding DNA in a sequence-specific manner and (2) mediating transcriptional activity (Lambert et al., 2018). EAF2 functions as a TF in both ways. First, the EAF2 protein could bind to DNA (a hexamer nucleotide sequence: ACTTTA) in a sequence-specific manner (Xiao et al., 2006). Second, multiple segments modulating transactivation have been reported. The amino acid sequence 177–262 in the C-terminus domain of EAF2 was originally reported by Simone et al. (2003). Subsequently, Xiao et al. reported four domains (amino acid sequences 36–67, 68–113, 162–245, and 246–262) that could enhance transactivation and one (amino acid sequence 1–35) that could repress transactivation (Xiao et al., 2006). In addition, EAF2 also binds to or interacts with other proteins to indirectly mediate transcriptional activity.

### 4.1 Interaction with ELL

Since EAF2 was identified, ELL has emerged as its most well-known binding partner (Simone et al., 2003). ELL is a fusion gene of MLL resulting from the t (11; 19) (q23; p13.1) chromosomal translocation in acute myeloid leukemia, and it was named for its highly basic lysine-rich motif (Thirman et al., 1994). The ELL protein functions as an elongation factor that can enhance the catalytic rate of RNA polymerase II (Shilatifard et al., 1996) by suppressing the transient pausing of transcription elongation (Gerber et al., 2001). Simone et al. observed that EAF2 co-localizes with ELL into nuclear speckles, which facilitates EAF2 to control transcriptional activity more effectively (Simone et al., 2003). A subsequent study further investigated the association between EAF2 and ELL, finding that a type of binding between ELL and EAF2 was required to form nuclear speckles, and this binding could enhance the transactivation activity of EAF2 (Xiao et al., 2006). In addition, the presence of ELL may further enhance EAF2 stability, likely due to the fact that ELL1 can inhibit ubiquitination-dependent degradation of EAF2 (Yu et al., 2016).

Amino acids 36–133 of EAF2 were shown to be necessary to form nuclear speckles (Xiao et al., 2006). However, the results of the relocalization experiment by Zhuang et al. showed two differences from those of a previous study (Zhuang et al., 2008). First, amino acids 163–262, but not 36–133, were responsible for targeting EAF2 to nuclear speckles. They proposed that this discrepancy could be attributed to the difference between human and mouse EAF2 sequences. Second, EAF2 formation from nuclear speckles did not require the physical binding of ELL, indicating that ELL is an inducer in the process.

### 4.2 Wnt/β-catenin signaling

Another study proposed that EAF2 and EAF1 are components of the β-catenin transcriptional complex (Wise et al., 2017). Observations of zebrafish embryos suggested that EAF2/1 bind to β-catenin and other β-catenin transcriptional complexes to suppress Wnt/β-catenin signaling. The N-terminal region of EAF2 (amino acids 1–112) is responsible for the binding to β-catenin. In the patterning of the embryonic anterior neuroectoderm and mesoderm, EAF2 and EAF1 function as transcriptional suppressors to ensure normal development, and both the N- and C-termini of EAF2/1 must be intact to function as a suppressor, indicating the suppressive activity of EAF2 plays a key role in embryogenesis through Wnt/β-catenin signaling.

### 4.3 EAF2 suppresses the transcriptional activity of HIF-1α

HIF-1α is an important microenvironmental factor implicated in tumor initiation, progression, angiogenesis, metabolism, and invasion (Tsai and Wu, 2012). Chen et al. first found that EAF2 binds to HIF-1α through amino acids 36–49 and later successfully found that EAF2 is capable of inhibiting the transactivity of HIF-1α (Chen et al., 2014). The effect of EAF2 on the transcriptional activity of HIF-1α is a part of a negative feedback loop. EAF2 expression is stimulated by HIF-1α in hypoxic conditions; afterwards, overexpressed EAF2 binds to HIF-1α and inhibits its transitional activity by blocking the recruitment of the transcriptional coactivator CBP/p300. This may explain part of the function of EAF2 as a tumor suppressor.

## 5 Function of EAF2 in embryonic development

Shortly after the initial report on EAF2 in 2003, Li et al. determined the expression pattern of EAF2 during embryonic development by treating cryosections of mouse embryos with *in situ* hybridization. They found that EAF2 was preferentially expressed in the brain, spinal cord, cranial ganglia, spinal ganglia, developing otocysts, retina, and pituitary gland, suggesting that EAF2 may play a pivotal role in regulating the growth and differentiation of the nervous system. Furthermore, EAF2 transcription has been detected in organs and tissues, and its formation and development require active epithelium–mesenchymal interactions, including tooth buds, mammary glands, submandibular glands, pancreas, kidney, and lungs (Li et al., 2003).

Liu et al. explored the mechanisms of EAF2 in embryogenesis using animal models and provided two pieces of evidence to prove that EAF2 affects embryogenesis through non-canonical Wnt/β-catenin signaling: (1) EAF2/1 govern convergence and extension movements by modulating the expression of two non-canonical Wnt ligands, Wnt5 and Wnt11, which converge on a downstream gene, RhoA, to complete signal transduction (Lumetti et al., 2014; Liu et al., 2009) and (2) EAF2 and Wnt4, another non-canonical Wnt signaling ligand, form an autoregulatory negative feedback loop and modulate eye development in *Xenopus laevis* (Wan et al., 2010). Then, their focus turned to canonical Wnt/β-catenin signaling. In zebrafish embryos, EAF2/1 antagonize canonical Wnt/β-catenin signaling to modulate mesodermal and neural patterning processes (Liu et al., 2013). The suppressive effect of EAF2 on Wnt/β-catenin signaling was successfully confirmed in humans.

## 6 EAF2 in prostate cancer and other malignant diseases

### 6.1 EAF2 synergizes with PTEN and activates Akt and p44/42 pathways

EAF2 is an androgen-responsive tumor suppressor in the prostate, and it interacts with multiple genes via different pathways to influence prostate cancer (Xiao et al., 2003). One representative gene is *PTEN*, which encodes a major lipid phosphatase. Lipid phosphatase signals downstream through the Akt pathway to control proliferation, apoptosis, cell cycle, polarity, metabolism, migration, and angiogenesis. Mutation and deficiency of the *PTEN* gene are associated with multiple human tumors, including prostate cancer (Wise et al., 2017; Bedolla et al., 2007). Ai et al. observed that EAF2 and Pten double-deficient (EAF2^−/−^Pten^+/−^) mice exhibited a higher rate of prostate cancer compared with animals with EAF2 or PTEN deletion alone, and concurrent EAF2 and PTEN deficiency activates Akt and p44/42 signaling pathways (Ai et al., 2014). After castration of EAF2/Pten+/mice, an intense pAkt staining pattern was still sustained in wide prostatic tissues, suggesting that EAF2 and PTEN double deficiency may lead to castration resistance. The findings in mice have yet to be validated in human studies. According to their patient data, EAF2 and PTEN are downregulated concurrently in >50% of advanced human prostate cancer (Gleason scores of 8–9) specimens, indicating that EAF2 and PTEN co-downregulation represents poor prognosis (Ai et al., 2014). However, that their analysis was limited to prostate cancer specimens from only 20 patients. A large-scale clinical study is still needed to determine the effect of EAF2-PTEN interactions on castration resistance and prostate cancer prognosis.

### 6.2 EAF2 is associated with VHL and modulates the pVHL pathway

VHL is an important tumor suppressor and plays a key role in cellular oxygen sensing by targeting HIF (Chittiboina and Lonser, 2015). Xiao et al. reported that both EAF2- and VHL-knockout mice display abnormalities in angiogenesis and spermatogenesis, suggesting an association between these two genes (Xiao et al., 2009). Structurally, pVHL with intact alpha and beta domains is capable of binding directly to the N-terminus (amino acids 1–113) of EAF2. This binding could enhance pVHL stability and prolong its half-life (Xiao et al., 2009). Functionally, the associations between *VHL* and *EAF2* are two-fold. EAF2 deficiency reduces the level of pVHL, which is caused by the stabilizing function of direct binding between EAF2 and pVHL rather than the effect of EAF2 on pVHL transcription. Reduction of pVHL induced by EAF2 knockdown leads to enhanced angiogenesis (Xiao et al., 2009). In addition, VHL and EAF2 cooperate to regulate angiogenesis in the prostate. EAF2 and VHL double deficiency (EAF2^−/−^VHL^+/−^) mice exhibited increased vascular density compared to the control groups (wild-type, EAF2^−/−^ and VHL^+/−^mice), thus resulting in a higher incidence of prostatic intraepithelial neoplasia and stromal inflammation. The mechanism of elevated angiogenesis, irrespective of whether it is induced by EAF2 and VHL double deficiency or EAF2 deletion alone, lies in the VHL-HIF-vascular endothelial growth factor (VEGF) pathway. Reduction of pVHL enhances HIF-1α level, and the transcriptional activity of VEGF, a HIF target, will be subsequently stimulated. In expression profile of human prostate cancer tissue, the concurrent downregulation of EAF2 and upregulation of VEGF were in accord with the results observed in mice.

The relationship between EAF2 and VHL may help complete the HIF-1α suppressive function of EAF2. Under normoxia or atmospheric O_2_, EAF2 binds to and stabilizes pVHL, thus enhancing proteasomal degradation of HIF-1α, blocking HIF-driven angiogenesis. Under hypoxia, EAF2 expression is stimulated by HIF-1α, and according to the negative feedback loop, transactivity of HIF-1α will be next suppressed, protecting prostate cells against tumorigenesis (Chen et al., 2014).

### 6.3 EAF2 is associated with the ERK signaling pathway

Activation of the RAS-BRAF-MEK-ERK signaling pathway is related to the proliferation and growth of a broad range of human tumors (Maurer et al., 2011). The expression of molecules belonging to the ERK signaling pathway is elevated in patients with prostate cancer (Gioeli et al., 1999). Combined with the above two facts, Su et al. used bioinformatic analysis and cDNA microarrays and found that members of the RAS-ERK cascade, such as Grb2, PI3K, and BRAF, are upregulated in EAF2-deficiency murine models and that phosphorylated ERK (p-ERK) increases significantly (Su et al., 2013). The study of Su et al. validated the involvement of p-ERK in prostate tumorigenesis and demonstrated that the RAS-BRAF-MEK-ERK pathway is a downstream target of EAF2.

### 6.4 The function of EAF2 is related to the p53 gene in prostate cancer

As a pivotal tumor suppressor, *p53* is the most commonly mutated gene in human cancers (Olivier et al., 2004). Because ELL and VHL were both reported to be associated with p53 and EAF2 (Shinobu et al., 1999; Roe et al., 2006), there is a possibility of a link between EAF2 and p53.

Su et al. originally proved this link structurally by observing that EAF2 and p53 co-immunoprecipitated and co-localized in transfected cells (Su et al., 2010). Pascal and Wang subsequently determined that amino acids 245–262 of the C-terminal domain of EAF2 are essential for interaction with p53 by performing deletion mutagenesis on the EAF2 fragment (Wang et al., 2017). Functionally, EAF2 and p53 are associated in two aspects. First, they are involved in modulating the expression of thrombospondin-1 (TSP-1), a tumor suppressor and an antiangiogenic regulator. p53 suppresses the activity of the TSP-1 promoter and downregulates the expression of TSP-1 in prostate cancer cells (Kwak et al., 2002); however, this suppression would be significantly alleviated by co-transfection of EAF2 with p53 in PC-3 cells. In LNCaP and H1299 cell lines, except for lifting the suppression of TSP-1, EAF2 and p53 co-transfection could further increase TSP-1 promoter activity. Second, EAF2 and p53 are related through the STAT3 pathway. In LNCaP and C4-2, two human prostate cancer cell lines, the expression of STAT3 protein is not affected in response to individual knockdown of EAF2 or p53 but significantly increases after EAF2 and p53 double-knockdown; conversely, although double deficiency of EAF2 and p53 elevates proliferation in C4-2 cells, it is suspended by knockdown of STAT3. Besides, an increase in the expression of the STAT3 target genes, *CCL5*, *CXCL10*, and *GBP2*, in response to the combined knockdown was also detected. These findings indicate that EAF2 and p53 cooperate in suppressing STAT3 signaling and protecting the prostate from tumorigenesis (Wang et al., 2017). Therefore, therapeutic agents targeting STAT3 may be promising for prostate cancer patients exhibiting EAF2 deficiency.

### 6.5 EAF2 interacts with FOXA1

*FOXA1* drives prostate cancer initiation and progression. It is also a key member of the AR signaling pathway by regulating the transcription of downstream AR genes (Teng et al., 2021). Guo et al. first proved that EAF2 and FOXA1 could interact with each other in coimmunoprecipitation through amino acid sequences 1–113 of EAF2. Then, based on the results observed in LNCaP cells, they found that (1) EAF2 knockdown elevated FOXA1 level, stimulated expression of AR-targeted genes, and promoted tumor proliferation and invasion, (2) FOXA1 knockdown inhibited the expression of AR-targeted genes and tumor proliferation, and (3) the joint knockdown of EAF2 and FOXA1 slightly increased AR-targeted gene expression and tumor proliferation. Moreover, they claimed that the tumor-suppressive function of EAF2 is partly mediated through interaction with FOXA1 (Guo et al., 2015).

### 6.6 EAF2 in other malignant tumors

In addition to prostate cancer, EAF2 is associated with other tumors and malignancies. EAF2-null and -heterozygous mice developed a higher frequency of hepatocellular carcinoma, lung adenocarcinoma, and B-cell lymphoma compared to wild-type mice (Xiao et al., 2008). The association between EAF2 and hepatocellular carcinoma can be explained by the fact that EAF2 deficiency increases the risk of hepatic vascular lesions, which subsequently leads to hepatocyte loss, hepatic fibrosis, and eventually hepatocellular carcinoma via the pVHL pathway in mice (Pascal et al., 2011). However, the mechanism underlying the relationship between EAF2, lung adenocarcinoma, and B-cell lymphoma remains to be explored.

In 2019, a single-strand conformation polymorphism assay identified intratumor heterogeneity of EAF2 frameshift mutations in colorectal cancer (CRC) and the inactivation of EAF2 in microsatellite instability-high CRC (Jo et al., 2019). This finding prompted further research into the role and mechanism of EAF2 in CRC (Feng et al., 2022). Clinically, decreased expression of EAF2 was observed in CRC tissue, especially in advanced tumors, which was consistent with follow-up results showing that patients with low expression of EAF2 tend to have poorer survival rates. Biologically, research on human CRC cells *in vitro* has yielded three key findings: (1) overexpression of EAF2 suppresses the invasion and migration of CRC cells, (2) reduces the activity of STAT3/TGF-β1 crosstalk pathway, and (3) inhibits CRC angiogenesis. Based on these findings, Feng et al. confirmed that EAF2 mediates CRC angiogenesis via the STAT3/TGF-β1 pathway (Feng et al., 2022).

Sun et al. revealed an association between EAF2 and non-small cell lung cancer (NSCLC). First, they observed that the expression level of LINC00301, a nuclear long non-coding RNA (lncRNA) related to multiple human cancers (Lin et al., 2017), increases in both NSCLC cell lines and tumorous tissues (Sun et al., 2020). Subsequently, they found that LINC00301 overexpression could repress EAF2 expression, which would facilitate NSCLC cell growth and invasion via the pVHL pathway. Specifically, the upstream transcription factor, FOXC1, upregulates the expression of LINC00301 by interacting with the protein “enhancer of zeste homolog 2”. LINC00301, in turn, silences the expression of EAF2, leading to abnormal angiogenesis via the downstream pVHL/HIF-1α/VEGF pathway.

A recent study found that EAF2 is overexpressed in skin cutaneous melanoma (SKCM) (Han and Shen, 2020). Interestingly, elevated levels of EAF2 are correlated with advanced stages and better prognosis. Furthermore, Han et al. suggested that this seemingly contradictory finding could be attributed to the presence of infiltrated immune cells rather than cancer cells in SKCM tissues, as EAF2 was highly expressed in B cells (Li et al., 2016).

## 7 Role of EAF2 in benign diseases

### 7.1 EAF2 in benign prostate hyperplasia (BPH)

Although BPH differs from prostate cancer in terms of its clinical characteristics, localization, and pathological processes, both are strongly associated with androgens (Madersbacher et al., 2019). To explore the effect of androgens on the development of BPH, a study was designed to measure and calculate the expression of androgen-responsive genes in BPH compared to adjacent normal tissue (O’Malley et al., 2009). Notably, *EAF2* and its binding partner, *ELL2*, were included in the genes investigated in their study. The results showed that the expression levels of EAF2 and ELL2 in BPH epithelial cell specimens increased by 2.2 and 6.0 folds, respectively, compared with those in adjacent normal epithelial cells.

Given that EAF2 inactivation and deficiency may lead to prostate cancer (Wang et al., 2017), the upregulated expression of EAF2 may be partly responsible for protecting hyperplastic prostate tissues from malignancy. EAF2 and ELL2, along with other androgen-responsive genes, likely have functional impacts on BPH pathogenesis.

### 7.2 EAF2 in kidney stone disease

The incidence of nephrolithiasis in males appears to be three times higher than that in females, indicating that androgens and AR might play an important role in the formation of kidney stones (Fang et al., 2017). In 2019, a research team found that among patients with nephrolithiasis, individuals with the GG allele at the rs7627468 variant of the calcium-sensing receptor (CASR) gene have lower pH levels in urine (Chen et al., 2019), which may explain the involvement of CASR in stone formation (Vezzoli et al., 2011). Using the GTEx portal, a project for building a gene expression bank, they found that rs7627468 influences the expression of EAF2 (Chen et al., 2019), indicating that EAF2 may indirectly affect stone formation, but the specific mechanism remains unclear.

## 8 EAF2-mediated apoptosis

### 8.1 EAF2 mediates apoptosis in prostate cancer cells

Apoptosis is a series of genetically pre-programmed events that result in the destruction of cell membranes and chromosome condensation. A previous study supported the role of ELL in the induction of programmed cell death (Johnstone et al., 2001). EAF2 and ELL co-localize into distinct nuclear speckles, and the two can bind via specific domains. These results indicate that EAF2 is associated with apoptosis via ELL. Following this, Xiao et al. transfected an EAF2 expression vector into human prostate cancer cells and found that the EAF2 protein induces efficient apoptosis in transfected cells (Xiao et al., 2003). However, in androgen-sensitive human prostate cancer cells, although EAF2 activated the apoptosis process, androgens protected the cells from death. In contrast, in androgen-insensitive prostate cancer cells, androgens did not inhibit apoptosis. These results led to the speculation that androgens protect against EAF2-induced apoptosis when ARs are present. They also observed that EAF2-induced apoptosis is sensitive to caspase inhibitors, suggesting that EAF2-induced apoptosis is a caspase-dependent process (Xiao et al., 2003). Structurally, amino acids 68–113 of EAF2 are sufficient to induce apoptosis (Hahn et al., 2007). Notably, amino acids 68–113 were among the multiple domains that modulated transactivation in their previous study (Xiao et al., 2009). Although Xiao et al. observed apoptosis in transfected cells, it should be noted that in the process of transfection, EAF2 beyond a physiological dose was applied to prostate cancer cells. Therefore, it is still uncertain whether EAF2 at a physiological dose could induce apoptosis.

### 8.2 EAF2 mediates apoptosis in other cells

Germinal center (GC) B cells represent a unique cell type that can produce high-affinity antibodies required for protective immunity (Young and Brink, 2021) and differentiate into plasma or memory B cells population (Victora et al., 2010; Finkin et al., 2019). Compared to other types of immune cells, EAF2 is much more expressed in GC B cells and plays the role of apoptosis inducer. GC B cells from EAF2 deficiency mice (EAF2^−/−^) exhibited significantly reduced cell death compared to wild-type mice, resulting in the accumulation of non-proliferating B cells, enlarged GCs, and a temporarily enhanced primary immune response at the early stage. However, over the long term, impaired apoptosis of GC B cells in aged EAF2^−/−^ mice leads to an abnormal accumulation of various autoantibodies in the absence of external stimuli, making EAF2^−/−^ mice more susceptible to collagen-induced arthritis (Li et al., 2016). This study illustrates that EAF2 plays an important role in maintaining immunological balance and self-tolerance by inducing apoptosis in GC B cells.

Feng et al. reported a different role of EAF2 as apoptosis suppressors in ocular tissues (Feng and Guo, 2018). They observed that the induced EAF2 expression alleviated apoptosis caused by hydrogen peroxide in human lens epithelial cells. In addition, the knockdown of Wnt3a significantly increased the overall percentage of EAF2-expressing lens epithelial cells undergoing apoptosis, demonstrating that EAF2 protects these cells from apoptosis by activating the Wnt3a signaling pathway (Feng and Guo, 2018).

## 9 EAF2 regulates DNA repair

As reported previously, EAF2 functions as a transcription factor, and ELL facilitates the relocalization of EAF2 into nuclear speckles. Similarly, several proteins that function in DNA repair can change their intracellular localization and form DNA repair foci in the nucleus to cope with DNA damage (Tembe and Henderson, 2007). The similarity between EAF2 and these DNA repair-related proteins motivated Zhuang et al. to investigate whether EAF2 plays a role in DNA repair. They confirmed their hypothesis when they observed that UV irradiation-induced DNA damage was fixed by EAF2 transfection, and amino acids 163–262 were subsequently identified as sufficient to induce EAF2 translocation to the nucleoli (Zhuang et al., 2008).

Subsequently, to explore the mechanism underlying the association between EAF2 and DNA damage response, Ai et al. treated human prostate cancer cells with irradiation to induce double-stranded breaks (DSBs). EAF2 and EAF1 co-accumulated rapidly at DSB regions with Ku70/Ku80, two non-homologous end-joining (NHEJ) pathway proteins. They further observed that the knockdown of EAF family proteins inhibits the accumulation of Ku70/Ku80 at DSBs. Both results demonstrated that the EAF family proteins work with Ku70/Ku80 to protect cells from DNA damage via the NHEJ pathway (Ai et al., 2017).

## 10 Discussion

**FIGURE 2:**
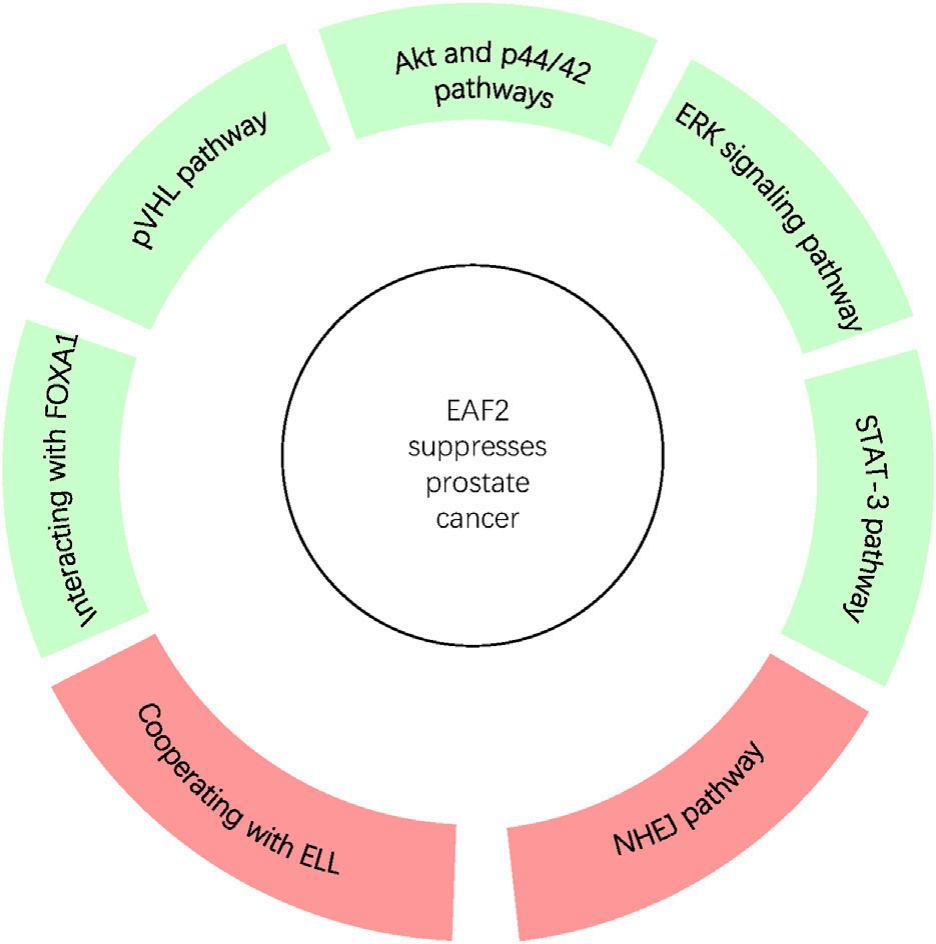
In prostate, EAF2 protects overall cellular health and suppresses tumorigenesis through coordination of interaction with proteins via various pathways. Green and Red blocks represent pathways and processes suppressed or activated by the expression of EAF2 respectively.

**FIGURE 3:**
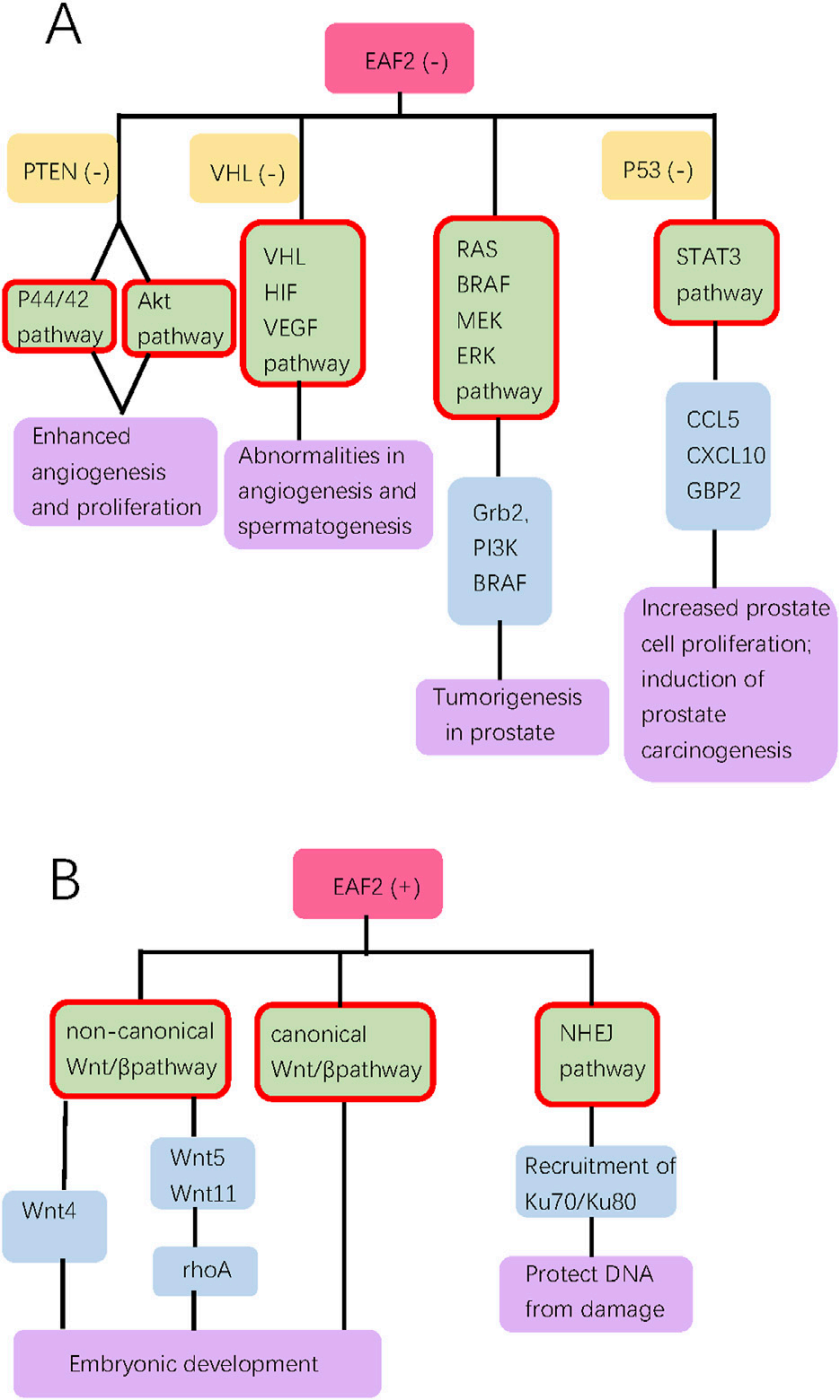
This diagram summarizes all the signal pathways discussed in the review, categorizing them into groups **(A, B)**. Groups A and B show pathways activated with EAF2 deficiency and presence, respectively. Pink, orange, green, blue, and purple bubbles indicate the state of EAF2, concurrent deficiency of another gene, pathways (highlighted with red border), downstream proteins, and biological effect of pathways, respectively.

Since EAF2 was identified, an increasing number of its functions and corresponding mechanisms have been explored. EAF2 affects the prostate and other organs. We have summarized the functions of EAF2 in transcription, embryonic development, malignant diseases, benign diseases, apoptosis, and DNA repair. In the prostate, the properties of EAF2 protein, its associations with multiple molecules, and its functions illustrate a high level of consistency. For instance, EAF2 is a nuclear protein, which aligns with its role in mediating transcriptional activity, inducing apoptosis, and repairing DNA damage. These functions are crucial in protecting prostate cells from tumorigenesis and uncontrolled cell growth. In Figure 1, Green blocks represent pathways or physiological processes enhanced by downregulation of EAF2, while red blocks represent processed activated by upregulation of EAF2. Multiple physiological functions of EAF2 coordinate to suppress tumorigenesis. Figures 2, 3 summarize the structural and functional characteristics of EAF2. As shown in Figure 2, different researches proved that different domains of EAF2 cross and cover each other structurally, which also confirmed that as a nuclear protein, the functions of apoptosis, DNA repair, and tumor suppression interrelate and contribute to cellular health jointly. Besides, it is reasonable that the region interacting with pVHL (amino acids 1–113) includes the region interacting with HIF-1α (amino acids 36–49), considering that both of them belong to the same signal channel to regulate angiogenesis. Figure 3 shows all the signaling pathways and their respective effects that are discussed in this review. We divided the pathways mentioned in the review into two groups: (A) group indicates pathways activated with EAF2 downregulation and (B) represents pathways activated when EAF2 is expressed.

The primary role of EAF2 is an androgen-upregulated tumor suppressor in the prostate. However, the detailed upstream pathways that regulate the expression of EAF2 remain unclear and require further exploration. Research in above mentioned directions will provide insights into the roles of androgens and EAF2 in prostate carcinogenesis, as well as opportunities to optimize chemical castration therapy for prostate cancer.
